# Access to healthcare among transgender women living with and without HIV in the United States: associations with gender minority stress and resilience factors

**DOI:** 10.1186/s12889-024-17764-y

**Published:** 2024-01-20

**Authors:** Talia A. Loeb, Sarah M. Murray, Erin E. Cooney, Tonia Poteat, Keri N. Althoff, Christopher M. Cannon, Jason S. Schneider, Kenneth H. Mayer, J. Sonya Haw, Andrew J. Wawrzyniak, Asa E. Radix, Jowanna Malone, Dee Adams, Megan Stevenson, Sari L. Reisner, Andrea L. Wirtz, American Cohort to Study HIV Acquisition Among Transgender Women Study Group

**Affiliations:** 1 Department of Epidemiology, Johns Hopkins Bloomberg School of Public Health, 615 N. Wolfe Street, E6014, Baltimore, MD 21205 USA; 2 Department of Mental Health, Johns Hopkins Bloomberg School of Public Health, Baltimore, MD USA; 3 Department of International Health, Johns Hopkins Bloomberg School of Public Health, Baltimore, MD USA; 4 Department of Social Medicine, University of North Carolina School of Medicine, Chapel Hill, NC USA; 5 Whitman-Walter Institute, Inc., Washington, DC USA; 6 Department of Medicine, Emory University School of Medicine, Atlanta, GA USA; 7 The Fenway Institute, Fenway Health, 1340 Boylston Street, 8th Floor, Boston, MA 02215 USA; 8 Beth Israel Deaconess Medical Center, Boston, MA USA; 9 Harvard Medical School, Boston, MA USA; 10 Division of Endocrinology, Metabolism and Lipids, Emory University School of Medicine, Atlanta, GA USA; 11 Department of Psychiatry and Behavioral Sciences, University of Miami Miller School of Medicine, Miami, FL USA; 12 Department of Medicine, Callen-Lorde Community Health Center, New York, NY USA; 13 Brigham and Women’s Hospital, Boston, MA USA; 14 Harvard School of Public Health, Boston, MA USA

**Keywords:** Transgender women, Gender minority stress, Resilience, Healthcare access, HIV, Structural equation modeling, Key populations

## Abstract

**Background:**

Transgender women (TW) experience significant inequities in healthcare access and health disparities compared to cisgender populations. Access to non-transition related healthcare is understudied among TW. We aimed to assess the association between access to care and gender minority stress and resilience factors among TW living with and without HIV in eastern and southern United States.

**Methods:**

This study was a cross-sectional analysis of baseline data drawn from a cohort of 1613 adult TW from the LITE Study. The cohort permitted participation through two modes: a site-based, technology-enhanced mode and an exclusively online (remote) mode. Exploratory and confirmatory factor analyses determined measurement models for gender minority stress, resilience, and healthcare access. Structural equation modeling was used to assess the relationships between these constructs. Models were evaluated within the overall sample and separately by mode and HIV status.

**Results:**

Higher levels of gender minority stress, as measured by anticipated discrimination and non-affirmation were associated with decreased access to healthcare. Among TW living with HIV, higher levels of anticipated discrimination, non-affirmation, and social support were associated with decreased healthcare access. Among TW living without HIV in the site-based mode, resilience was positively associated with positive healthcare experiences and inversely associated with barriers to healthcare access. Among TW living without HIV in the online mode, anticipated discrimination was associated with barriers to healthcare access; resilience was positively associated with positive healthcare experiences and inversely associated with barriers to healthcare access.

**Conclusions:**

Gender minority stress was associated with increased barriers to healthcare access among TW in the US, regardless of HIV status. Resilience factors did not mediate this effect. Interventions aiming to increase healthcare access among TW can be aided by efforts to mitigate drivers of gender minority stress and improve patient experiences in healthcare facilities.

**Supplementary Information:**

The online version contains supplementary material available at 10.1186/s12889-024-17764-y.

## Background

Transgender women (TW) in the United States (US) experience critical barriers to healthcare and significant health inequities due to their minoritized status. These inequities include lower rates of insurance coverage, and a disproportionate prevalence of HIV, depression, anxiety, lifetime suicide attempts, and illicit drug use compared to the general population [1]. They are also more likely to experience violence [2].

Transgender individuals experience chronic stress due to stigma and discrimination, which is associated with poor health outcomes [3], that are exacerbated by barriers to healthcare access [2]. The Affordable Care Act’s Sect. 1557 prohibits discrimination on the basis of sex for health entities, including insurance coverage. This includes gender identity and bans exclusions of transgender individuals [4]. However, as many as 25% of transgender individuals were still denied health care coverage because of their identity as of 2019 [1]. Despite these structural limitations to healthcare access, general health (i.e., overall health and health unrelated to gender affirming care) is considered one of the least researched aspects of transgender health [5, 6].

### Access to healthcare

We conceptualized healthcare access consistent with the Institute of Medicine Committee on Monitoring Access to Personal Health Care Services (1993) comprehensive framework [7]: access to healthcare relies on use of health services and achievement of health outcomes and multiple factors (e.g., structural, financial, personal, or cultural) can act as barriers. Utilization of healthcare is characterized by frequency and continuity of care (i.e., regular use of healthcare services), the type of provider (primary), the setting (location in which services are used), and the purpose, including prevention and treatment. Outcomes are characterized by survival and satisfaction of services utilized, both of which can include health status and the quality of care [7]. Recent studies primarily operationalize access to care as having insurance coverage, having a regular place for care, and affordable cost of care [8, 9], which are only partially consistent with the framework described above. Among TW, studies have characterized healthcare access as experiences of discrimination in the healthcare system. However, most existing studies have assessed access to gender-affirming or HIV-related care among TW, rather than to general healthcare [10].

### Gender minority stress theory

Minority Stress Theory characterizes stressors as contributors to poor mental and physical health for minoritized populations, while also describing resilience as a protective factor for health [11]. Stressors are categorized as either distal, i.e., external experiences such as exposure to discrimination and stigma, or proximal, i.e., internal experiences such as anticipatory stigma and concealment of identity [12]. Originally developed to describe stress experienced by sexual minorities [13], the framework has since been expanded to understand stressors associated with numerous intersections of marginalized identities [14, 15]. Specifically, Gender Minority Stress Theory focuses on the individual and social stressors and experiences of transgender persons, who, in comparison to their cisgender counterparts, face unique challenges and forms of discrimination such as differences between their gender identity and the gender recorded on legal documents, difficulty accessing safe public bathrooms, and other experiences of non-affirmation [16, 17].

Stigma and discrimination hinder access to and utilization of healthcare. Anticipatory stigma has been found to be associated with low utilization of gender-affirming healthcare [11]. Notably, experiences of stigma and discrimination can occur within the healthcare system and discourage or prevent TW from seeking further care due to the anticipatory stigma [18].

According to the Minority Stress Theory, resilience factors, defined as factors related to the ability to recover from and adapt to adversities [19], can modify the relationship between discrimination and health outcomes among minoritized groups [14]. Social support, community connectedness, and family support have been shown to be positively associated with resilience [19], and therefore could buffer the effects of discrimination and adverse health outcomes. For example, one study showed that transgender individuals who were connected to other people who identify as transgender reported less anxiety and suicidality, and another showed that people who were not connected to the community and experienced internalized transphobia experienced more adverse mental health outcomes [16].

TW also experience a disproportionately high burden of HIV, with an odds of HIV infection 48 to 66 times higher than that of other adult populations [20–22]. This inequity is attributed to systemic barriers and structural challenges, such as housing, employment, insurance, and legal recognition, which largely reflect gendered and societal structures, placing transgender people in vulnerable positions for various health conditions [6, 9]. However, people living with HIV have access to primary healthcare and support services through the Health Resources and Services Administration (HRSA) and the Ryan White HIV/AIDS Program in the United States [23].

### Objectives

Due to the lack of research on the associations between gender minority stress and resilience and access to healthcare among TW in the US, this study sought to investigate how gender minority stress and resilience factors are associated with access to care among a sample of TW in the US. We further evaluated whether the associations between gender minority stress, resilience factors, and access to healthcare among TW vary by HIV status.

## Methods

This study is a secondary analysis of baseline data collected in the Leading Innovation for TW’s Health and Empowerment (LITE) study, a prospective cohort study of TW in the United States that aims to investigate gaps in knowledge on HIV prevention and care for TW to inform interventions to address health inequities. Baseline data were collected at enrollment, between March 2018 and August 2020.

LITE’s study procedures have been previously described [24, 25]. We recruited participants through a mix of technology- and community-based methods. Once recruited, participants could participate in the cohort through two modes of participation: (1) site-based technology-enhanced mode in collaboration with research and clinical institutions in Atlanta, Baltimore, Boston, Miami, New York City, and Washington, DC, and (2) an exclusively digital (online) mode. The digital mode was geotargeted to 72 eastern and southern US cities that matched the original six cities based on population size and demographics. Modes of participation did not necessarily reflect the method of recruitment for each participant [24, 25]. At baseline, all participants completed a self-administered socio-behavioral survey and HIV/STI self-testing. Because the study was open to English- and Spanish-speaking participants, all materials were translated into Spanish, and Spanish-speaking staff were available to support participants.

The parent study has been approved by the Johns Hopkins School of Medicine Single Institutional Review Board. This secondary data analysis was approved by the Johns Hopkins Bloomberg School of Public Health Institutional Review Board.

### Participants

Participants were transgender adults (aged 18 or older) who were assigned male at birth but identified along the transfeminine spectrum, including the following identities: woman, transgender woman, nonbinary, or other gender diverse identity. Gender identity was verified at enrollment using a two-step method [26]. HIV status was verified using oral fluid HIV testing. In the site-based mode, participants who were living with HIV were eligible to participate in the baseline assessment; resource constraints prohibited inclusion of people living with HIV in the baseline assessment of the online mode [24, 25].

### Measures

The primary outcome was access to care. We analyzed predictors, including gender minority stress and resilience factors, which were measured using responses to multiple observed indicators. Potential confounders were age, education, employment status, race and ethnicity, and citizenship. All measures were self-reported.

#### Access to care

Access to healthcare was operationalized with self-reported information for three domains: barriers, utilization, and outcome. Key indicators for barriers included health insurance coverage and experienced challenges in accessing care. Participants were asked to report their insurance type; for analysis, we dichotomized responses into either being insured or being uninsured. Challenges to accessing general healthcare, operationalized in this analysis as healthcare unrelated to gender-affirmation or transition, were assessed by asking “What are some of the challenges you face when accessing healthcare?” with select-all responses that included time, transportation, safety, childcare, cost, no health coverage, inconvenient hours, mistreatment for being transgender, had bad experiences in the past, healthcare providers’ discomfort for caring for transgender patients, and other.

Utilization was assessed with a series of binary questions asking participants whether they had a primary care provider, a consistent facility to receive care, and a visit with a healthcare provider in the past year. Outcome measures for access to care were assessed through Likert scales for satisfaction, health status, and whether they felt their physician was knowledgeable about transgender issues. All access to care variables were transformed into binary indicators for inclusion in the model. Gender affirming care utilization was not measured in this analysis, as it was expected to conceptually overlap with affirmation/resilience and access to general healthcare.

#### Minority stress factors

Gender minority stress is generated directly through experiences of enacted discrimination, anticipated discrimination, or internalized discrimination related to gender identity [27]. The key indicators in the measurement model are direct measures of anticipated discrimination based on gender identity, non-affirmation of gender identity, and mental distress, measured as a binary indicator based on the CDC Healthy Days Measure [28]. Victimization (i.e., physical violence, sexual violence, psychological violence and trans-specific forms of intimate partner violence) was included in the structural model as a predictor of gender minority stress [29]. Mental distress [30] was included as an indicator of gender minority stress because the causal relationship identified between mental distress and gender minority stress in the framework suggests that mental distress can be a visible manifestation of the occurrence of stress. All victimization measures were assessed for lifetime and recent experiences (in the past 12 months). Sexual violence, physical violence, and intimate partner violence were weakly correlated, and thus, they were not correlated in the structural model.

Measures of discrimination were adapted from the Anticipated Discrimination subscale of the Intersectional Discrimination Scale [31], which consists of nine Likert scale items that assess the extent of participant agreement with various statements regarding interpersonal experiences on the basis of “who they are” rather than specifically attributed to gender identity.

Non-affirmation was measured by a validated measure that comprises a subscale of the Gender Minority Stress and Resilience measure [16]. This consists of six-items with a four-point Likert scale assessing agreement with the following statements: “I have to repeatedly explain my gender identity to people or correct the pronouns people use,” “I have difficulty being perceived as my gender,” “I have to work hard for people to see my gender accurately,” “I have to be extra feminine in order for people to accept my gender,” “People do not respect my gender identity because of my appearance or body,” and “People do not understand me because they do not see my gender as I do” [16].

#### Resilience factors

Resilience refers to individuals overcoming negative health consequences when encountering a stressor by utilizing coping mechanisms and resources [11]. The key indicators for resilience factors included pride, social support, family support, community connectedness, body comfort, and the extent to which their gender identity is expressed legally, based on the use of these factors in previous studies specific to resilience [12, 19]. However, pride and community connectedness measures were not included in the survey for the online mode and thus were not analytic variables for this group. Pride was measured using the Transgender Identity Survey- Pride Subscale, an eight-item measure with 3-point Likert scale responses ranging from disagree to agree [16, 32]. Social support was measured using the California Health Interview Survey Social Support Measure, a six-item measure with 5-point Likert scale responses ranging from none of the time to all of the time [33].

Community connectedness was measured using a validated a five-item Gender Minority Stress and Resilience- Community Connectedness measure, in which participants are asked to disagree or agree with statements using a 3-point Likert scale [16]. Body comfort measured using a 5-item Likert scale response to the question “How comfortable do you currently feel with your body”. This item was added at the recommendation of the study community advisory board. The extent to which one’s identity was legally affirmed was measured by two items evaluating the extent to which the participant’s name and preferred gender are listed on legal identifications, ranging from none to all.

Supplemental Table 1 details Cronbach’s alpha values for internal reliability of all scales.

### Statistical analysis

#### Exploratory data analysis

We explored patterns of missingness and generated descriptive statistics for all measures. None of the variables in the study had missingness greater than 10%. The question, “How knowledgeable do you feel the healthcare provider you’ve seen most recently is about health issues facing trans people?” had the greatest missingness at 9.3%. Individuals were more likely to be missing responses to this question if they reported receipt of care primarily in the hospital emergency room, did not have a regular source of healthcare, or had not seen a healthcare provider in the past year.

Exploratory analyses showed that there were significant differences in most demographic characteristics between transgender women living with HIV (TWLHIV) in the site-based mode, TW living without HIV in the site-based mode, and TW living without HIV in the online mode. This, along with increased access to support services for individuals living with HIV in the United States, may introduce differences in how access to healthcare is experienced by TW living with versus without HIV. For this reason, analyses were conducted for the overall sample (subsequently referred as the entire sample), as well as separately for these three participant groups (subsequently referred to as the analytic groups).

#### Exploratory and confirmatory factor analysis

Exploratory and confirmatory factor analyses (EFA and CFA, respectively) were conducted to identify the underlying psychometric structure and to reduce the number of items for each latent construct. The analytic groups were randomly split in half to form two datasets, one for developing the models using EFA and one for testing the measurement models using CFA. The assumption of multivariate normality was assessed by constructing histograms and conducting multivariate tests for normality. Because this assumption was violated for most variables, iterative principal factor estimation methods were used in factor analyses.

A principal components analysis was first conducted on a correlation matrix to determine the number of factors to include in the measurement model. For gender minority stress and resilience, polychoric correlation matrices were used, because all indicators were ordinal, whereas for access to care, tetrachoric correlation matrices were used, as all indicators were binary. The number of eigenvalues over 1, the percent of variance explained, parallel analysis, and an examination of scree plots were all considered in choosing the number of factors. An EFA was then performed using promax rotation if factors were correlated at greater than 0.3 or less than − 0.3, suggesting correlated factors, and otherwise using varimax rotation. The rotated factor loadings (loading greater than 0.4) and uniqueness values (uniqueness less than 0.5) were evaluated to determine whether an item was retained in the model. This process was repeated iteratively, removing any indicators with low factor loadings or high uniqueness values, until the factor loadings did not change. If there was evidence of correlated factors in the EFA, this was included in subsequent models.

Results from the EFAs dictated choice of measurement models for the CFAs. In the CFAs, model fit was assessed using the following indices: chi-square test (p-value > 0.05 considered adequate), root mean-square error of approximation (RMSEA) (< 0.08 considered adequate), standardized root mean square residual (SRMR) (< 0.08 considered adequate), Tucker-Lewis fit index (TLI) (> 0.95 considered good, > 0.90 considered acceptable), and the comparative fit index (CFI) (> 0.95 considered good, > 0.90 considered acceptable) [34]. All indicators and outcomes were standardized (stdyx).

#### Structural equation modeling

Structural equation modeling (SEM) was then used to simultaneously estimate the measurement models with the inclusion of structural model components. Because of measurement non-invariance between the analytic groups, the SEM was done separately for each analytic group. Unmediated and mediated models were developed and tested to assess the degree to which resilience mediates the association between gender minority stress and access to care based on our a priori hypothesis. Additionally, several iterations of the models were run including potential confounders, defined a priori, to determine best fit.

**Fig. 1:**
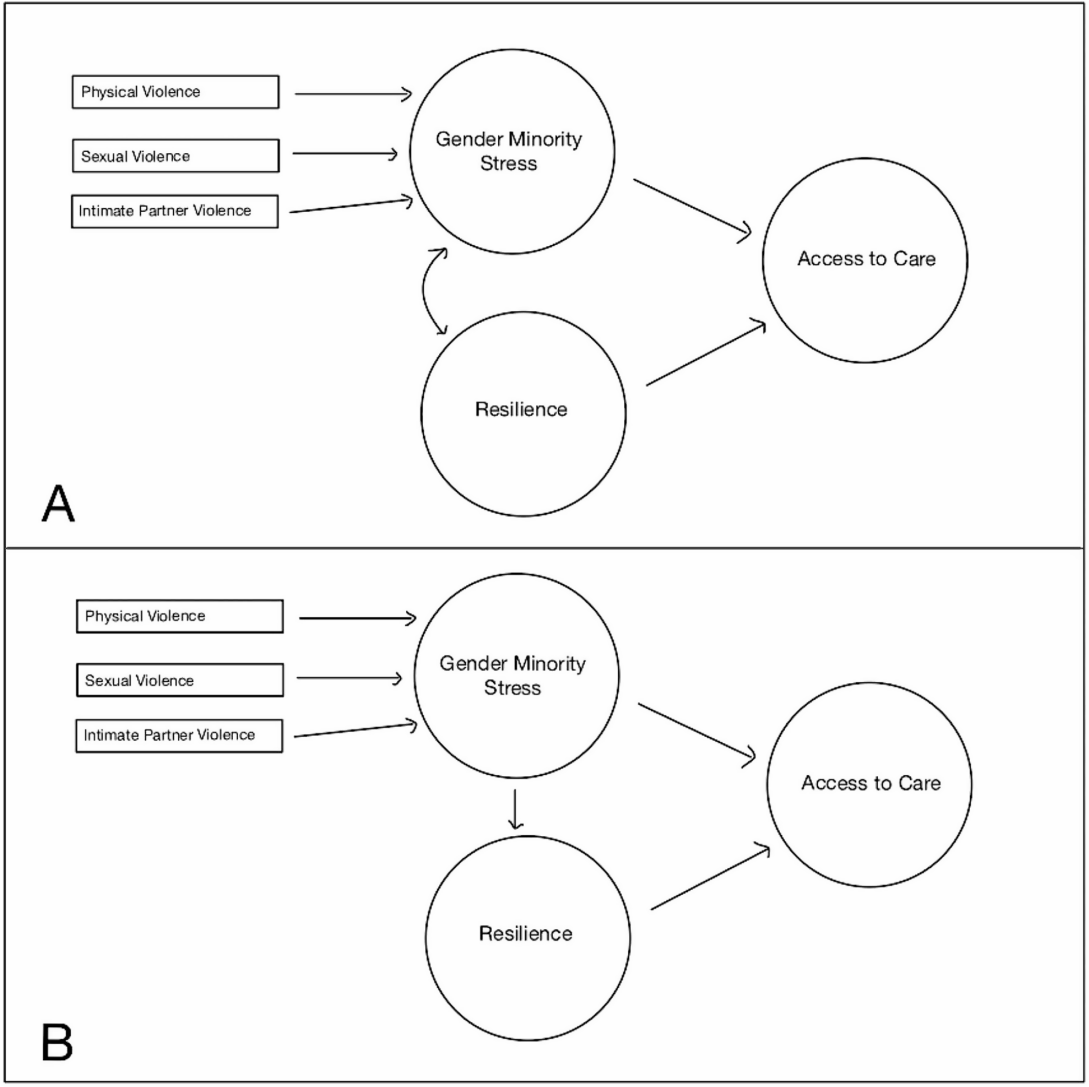
(**A**) Proposed unmediated structural model. (**B**) Proposed mediated structural model

SEM specification took into consideration minority stress theory and results of the EFA, CFA, and exploratory data analysis (Fig. 1a, b). Minority stress theory informed the hypothesized structural model, suggesting that gender minority stress would be associated with access to care, and that resilience may mediate this relationship. The model was fit using diagonally weighted least squares mean and variance adjusted estimators to accommodate ordinal data. We examined model fit using the same indices and values used for CFA.

Exploratory data analysis and EFA were conducted using Stata version 17 [35], and CFA and SEM were conducted using MPlus version 8.7 Base Program [36].

## Results

A total of 1,615 TW contributed baseline data across cohorts. Two participants were missing information on HIV status and were excluded from the analysis; thus, the analytic sample included 1,613 participants (Tables 1 and 2). Participants enrolled in online and site-based modes were different across various demographic characteristics.

**Table 1:** Demographic characteristics of transgender women in Eastern and Southern US overall and by HIV status and mode (*N* = 1613)

	TW Living with HIV (*n* = 280)	TW Living without HIV, Site-Based (*n* = 749)	TW Living without HIV, Online (*n* = 584)	Total (*N* = 1613)	p-value**
	n (%)	n (%)	n (%)	n (%)	
Age (median, IQR)	40 (31–51)	29 (24–38)	27 (22–34)	30 (24–39)	< 0.05
Education					< 0.05
High School or Less Some College or Higher Unknown	180 (64.3) 98 (35.0) 2 (0.7)	261 (34.9) 481 (64.2) 7 (0.9)	106 (18.2) 473 (81.1) 4 (0.7)	547 (33.9) 1052 (65.3) 13 (0.8)	
Employment					< 0.05
Unemployed Employed Unknown	212 (75.7) 64 (22.9) 4 (1.4)	337 (45.0) 394 (52.6) 18 (2.4)	198 (34.0) 364 (62.4) 21 (3.6)	747 (46.3) 822 (51.0) 43 (2.7)	
Race					< 0.05
Non-Hispanic White Non-Hispanic Black Hispanic White Hispanic Black Non-Hispanic Multiracial Hispanic Multiracial Unknown	12 (4.3) 153 (54.6) 24 (8.6) 11 (3.9) 36 (12.9) 41 (14.6) 3 (1.1)	274 (36.6) 148 (19.8) 68 (9.1) 22 (2.9) 113 (15.1) 113 (15.1) 11 (1.5)	432 (74.0) 28 (4.8) 19 (3.2) 1 (0.2) 74 (12.7) 23 (3.9) 7 (1.2)	718 (44.5) 329 (20.4) 111 (6.9) 34 (2.1) 223 (13.8) 177 (11.0) 21 (1.3)	
Citizenship					< 0.05
US Citizen Not a US Citizen Prefer Not to Answer	245 (87.5) 32 (11.4) 3 (1.1)	666 (88.9) 82 (11.0) 1 (0.1)	575 (98.6) 7 (1.2) 1 (0.2)	1486 (92.2) 121 (7.5) 5 (0.3)	
Live in a Medicaid Expansion State^+^	168 (60.0)	590 (78.8)	415 (71.1)	1173 (72.7)	< 0.05
Pre-Exposure Prophylaxis					
Ever Taken PrEP	17 (7.91)	211 (33.8)	40 (10.2)	268 (21.8)	< 0.05
Currently Taking PrEP*	-	124 (58.2)	24 (58.5)	149 (58.0)	0.876
HIV Care Continuum (*n* = 223)		-	-	-	-
Aware of Positive Status^−−^ Linked to Care^−^(*n* = 212) On ART (*n* = 193) Virally Suppressed (*n* = 190)	220 (98.65) 207 (97.64) 186 (96.37) 155 (81.58)				
Gender Affirming Care					
Tried to get in the past 12 months (*n* = 1609)	201 (72.0)	638 (85.3)	463 (79.6)	1302 (80.9)	< 0.05
Able to get Affirming Care	163 (58.2)	570 (76.1)	406 (69.5)	1139 (70.6)	< 0.05
Ever Had Hormone Treatment	206 (73.8)	661 (88.4)	444 (76.3)	1311 (81.5)	< 0.05
Taken Hormones in the past 6 months (*n* = 736) Ever received counseling	1 (1.4) 177 (63.4)	0 (0) 552 (73.8)	426 (73.6) 433 (74.4)	427 (58.0) 1162 (72.2)	< 0.05 < 0.05
Received counseling or therapy in the past 6 months (*n* = 881)	0 (0.0)	0 (0.0)	280 (48.3)	280 (31.8)	< 0.05

^+^Medicaid expansion was determined by data from the Kaiser Family Foundation [48]

*among those that report ever taking PrEP

**from Pearson chi-squared for categorical variables and ANOVA for continuous variables

--Aware of status is characterized by individuals who reported their most recent HIV test as positive and tested positive at baseline

^−^Linked to Care is characterized by individuals who have seen an HIV care provider in the previous 12 months

**Table 2:** Gender minority stress, resilience, and access to healthcare variables among transgender women in Eastern and Southern US overall and by HIV status and mode

	TW Living with HIV (*n* = 280)	TW Living without HIV, Site-Based (*n* = 749)	TW Living without HIV, Online (*n* = 584)	Total (*N* = 1613)	p-value**
	n (%) or median (IQR)	n (%) or median (IQR)	n (%) or median (IQR)	n (%) or median (IQR)	
**Gender Minority Stress**					
Non-affirmation	9 (5–14)	10 (5–15)	13 (9–17)	11 (6–15)	< 0.05
Physical Violence, ever	152 (54.7)	466 (62.3)	390 (67.1)	1008 (62.7)	< 0.05
Sexual Violence, ever	112 (40.3)	324 (43.3)	240 (41.3)	676 (42.1)	< 0.05
Intimate Partner Violence, past 12 months	0 (0–1)	0 (0–1)	0 (0–1)	0 (0–1)	< 0.05
Discrimination	15.5 (9-21.5)	21 (13–27)	24 (19–28)	21 (15–27)	< 0.05
**Resilience**					
Pride	15 (11–16)	12 (8–15)	-	13 (9–16)	< 0.05
Community Connectedness	6 (5–8)	7 (5–9)	-	6 (5–8)	< 0.05
Social Support	10 (6–15)	12 (7–16)	11 (7–16)	11 (7–16)	0.1070
Body Comfort					< 0.05
Very Uncomfortable Uncomfortable Neither Comfortable nor Uncomfortable	65 (23.3) 27 (9.7) 46 (16.5)	133 (17.8) 147 (19.6) 121 (16.2)	125 (21.5) 205 (35.2) 109 (18.7)	324 (20.1) 380 (23.6) 276 (17.1)	
Comfortable Very Comfortable Prefer not to answer	55 (19.7) 82 (29.4) 4 (1.4)	219 (29.3) 118 (15.8) 10 (1.3)	117 (20.1) 26 (4.5) 0 (0)	391 (24.3) 226 (14.0) 14 (0.9)	
Family Support					< 0.05
Strongly Disagree Disagree Neither agree nor disagree Agree Strongly agree Prefer not to answer	27 (9.7) 19 (6.8) 45 (16.1) 78 (28.0) 91 (32.6) 19 (6.8)	118 (15.8) 89 (11.9) 145 (19.4) 197 (26.3) 180 (24.1) 19 (2.5)	114 (19.6) 77 (13.2) 147 (25.3) 153 (25.3) 75 (12.9) 16 (2.7)	259 (16.1) 185 (11.5) 338 (21.0) 429 (26.6) 346 (21.5) 54 (3.3)	
**Access to Healthcare**					
Challenges to Access					
Time Transportation Worried about Safety Childcare Cost No Health Coverage Hours Not Convenient Mistreatment by Staff Bad Experiences in the Past Providers not comfortable caring for transgender patients	106 (37.9) 141 (50.4) 82 (29.3) 1 (0.4) 84 (30.0) 43 (15.4) 55 (19.6) 35 (12.5) 84 (30.0) 54 (19.3)	343 (45.8) 306 (40.8) 155 (20.7) 10 (1.3) 361 (48.2) 170 (22.7) 199 (26.6) 163 (21.8) 281 (37.5) 243 (32.4)	338 (58.1) 226 (38.8) 120 (20.6) 22 (3.8) 371 (63.7) 164 (28.2) 229 (39.3) 183 (31.4) 256 (44.0) 283 (48.6)	788 (48.8) 675 (41.8) 357 (22.1) 33 (2.0) 818 (50.1) 377 (23.4) 484 (30.0) 381 (23.6) 621 (38.5) 580 (36.0)	< 0.05 < 0.05 < 0.05 < 0.05 < 0.05 < 0.05 < 0.05 < 0.05 < 0.05 < 0.05
Health Insurance					< 0.05
Uninsured Public Insurance Private Insurance Unknown	12 (4.3) 246 (87.9) 8 (2.9) 14 (5.0)	71 (9.5) 360 (48.1) 266 (35.5) 52 (6.9)	54 (9.2) 151 (26.0) 349 (60.0) 28 (4.8)	137 (8.5) 758 (47.0) 624 (38.7) 94 (5.8)	
Has a Personal Healthcare Provider	231 (82.5)	572 (76.4)	366 (62.9)	1170 (72.5)	< 0.05
Satisfaction with Care					< 0.05
Dissatisfied Neither Satisfied nor Dissatisfied Satisfied Prefer not to answer	13 (4.8) 22 (8.1) 231 (84.9) 6 (2.2)	22 (3.1) 105 (15.0) 567 (80.9) 7 (1.0)	48 (9.3) 115 (22.2) 349 (67.5) 5 (1.0)	83 (5.6) 242 (16.2) 1149 (77.0) 18 (1.2)	
Provider Knowledgeable about Issues Facing Trans People? Not Knowledgeable Somewhat Knowledgeable Very Knowledgeable Prefer not to answer Don’t Know	24 (8.6) 42 (15.0) 187 (66.8) 11 (3.9) 16 (5.7)	47 (6.3) 153 (20.4) 475 (63.4) 7 (0.9) 67 (9.0)	117 (20.1) 175 (30.1) 222 (38.4) 2 (0.3) 66 (11.4)	188 (11.7) 371 (23.00) 885 (54.9) 20 (1.2) 149 (9.2)	< 0.05
Health Status					< 0.05
Poor Fair Good Very Good Excellent Prefer not to answer	7 (2.5) 48 (17.1) 76 (27.1) 65 (23.2) 79 (28.2) 5 (1.8)	23 (3.1) 114 (15.2) 230 (30.7) 222 (29.6) 157 (21.0) 3 (0.4)	36 (6.2) 132 (22.7) 188 (32.3) 169 (29.0) 56 (9.6) 1 (0.2)	66 (4.1) 294 (18.2) 494 (30.6) 458 (28.4) 292 (18.1) 9 (0.6)	

**From Pearson chi-squared for categorical variables and ANOVA for continuous variables

### Measurement model

Measurement models were fit for the entire sample and each of the three analytic groups (Table 3). Model fit tests indicated satisfactory fit except for resilience for the entire sample and access to healthcare among the online group that was living without HIV (Supplemental Table 3). Indicators included in the final measurement models explained at least 60% of the variance for each construct. Across all analytic groups, gender minority stress consisted of two factors: anticipated discrimination and non-affirmation. For the entire sample, resilience consisted of three factors (pride, social support, and community connectedness) and access to care consisted of three factors (treatment barriers, logistical barriers, and positive healthcare experiences). Among the TW Living with HIV, resilience consisted of three factors (pride, social support, and legal recognition) and access consisted of one factor (access barriers, or barriers to healthcare access). Among the site-based group that was living without HIV, resilience consisted of four factors (pride, community connectedness, social support, and legal recognition), and access to care consisted of two factors (positive healthcare experiences and barriers to healthcare access).

**Table 3:** Measurement model: standardized item loadings (standard error) for gender minority stress, resilience, and access to care factors

	Entire Sample^1^	TWLHIV^1~^	TW Living Without HIV, Site-Based^1^	TW Living Without HIV, Online^1+^^
**Anticipated Discrimination**				
Healthcare provider may treat me poorly	0.774 (0.016)	0.721 (0.042)	0.772 (0.024)	0.750 (0.029)
I might have trouble finding or keeping a job	0.828 (0.013)	0.835 (0.026)	0.803 (0.022)	0.828 (0.023)
I might have trouble getting an apartment or house	0.836 (0.012)	0.809 (0.028)	0.863 (0.015)	0.783 (0.023)
I worry about being treated unfairly by a teacher, supervisor, or employer.	0.841 (0.012)	0.863 (0.024)	0.831 (0.019)	0.807 (0.027)
I may be denied a bank account, loan, or mortgage	0.760 (0.015)	0.699 (0.043)	0.795 (0.020)	0.702 (0.032)
I worry about being harassed or stopped by police or security.	0.810 (0.014)	0.839 (0.028)	0.823 (0.020)	0.732 (0.031)
People might try to attack me physically	0.818 (0.014)	0.822 (0.033)	0.840 (0.018)	0.748 (0.031)
I expect to be pointed at, called names, or harassed when in public.	0.768 (0.016)	0.799 (0.035)	0.781 (0.023)	0.735 (0.029)
I fear that I will have a hard time finding friendship or romance.	---	0.713 (0.042)	---	---
**Non-affirmation**				
I have to repeatedly explain my gender identity to people or correct the pronouns people use.	0.699 (0.021)	---	0.760 (0.025)	0.647 (0.040)
I have difficulty being perceived as my gender.	0.840 (0.013)	0.838 (0.027)	0.854 (0.019)	0.792 (0.026)
I have to be extra feminine in order for people to accept my gender.	0.793 (0.016)	0.822 (0.031)	0.774 (0.023)	0.757 (0.032)
People do not respect my gender identity because of my appearance or body.	0.925 (0.009)	0.963 (0.013)	0.908 (0.015)	0.929 (0.015)
People do not understand me because they do not see my gender as I do.	0.888 (0.011)	0.0896 (0.021)	0.872 (0.017)	0.888 (0.019)
**Pride**				
My gender identity or expression makes me feel special and unique.	0.712 (0.033)	0.684 (0.075)	0.709 (0.038)	---
It is okay for me to have people know that my gender identity is different from my sex assigned at birth.	0.760 (0.028)	0.742 (0.056)	0.754 (0.033)	---
I have no problem talking about my gender identity and history to almost anyone.	0.786 (0.026)	0.693 (0.063)	0.790 (0.029)	---
It is a gift that my gender identity is different from my sex assigned at birth.	0.828 (0.023)	0.771 (0.054)	0.824 (0.027)	---
I am like other people but I am also special because my gender identity is different from my sex assigned at birth.	0.807 (0.024)	0.800 (0.046)	0.785 (0.030)	---
I am proud to be a person whose gender identity is different from my sex assigned at birth.	0.789 (0.025)	0.692 (0.070)	0.819 (0.028)	---
I am comfortable revealing to others that my gender identity is different from my sex assigned at birth.	0.886 (0.019)	0.816 (0.048)	0.879 (0.022)	---
I’d rather have people know everything and accept me with my gender identity and history.	0.703 (0.034)	0.668 (0.077)	0.708 (0.039)	---
**Community Connectedness**				
I feel part of a community with people who share my gender identity	0.900 (0.019)	0.890 (0.027)	0.935 (0.025)	---
I feel connected to other people who share my gender identity	0.915 (0.021)	0.848 (0.037)	0.913 (0.025)	---
When interacting with members of the community that shares my gender identity, I feel like I belong.	0.849 (0.023)	0.849 (0.036)	0.841 (0.031)	---
I am not like other people who share my gender identity.	-0.285 (0.053)		---	---
I feel isolated and separate from other people who share my gender identity.	-0.541 (0.047)		-0.413 (0.062)	---
**Social Support**				
Had someone to help take care of me when sick.	0.779 (0.016)	0.816 (0.034)	0.802 (0.022)	0.725 (0.033)
Had someone available to get together for relaxation.	0.887 (0.010)	0.914 (0.019)	0.913 (0.013)	0.828 (0.026)
Had someone available to understand your problems	0.895 (0.010)	0.975 (0.009)	0.908 (0.013)	0.805 (0.027)
Had someone available to love me and make me feel wanted.	0.873 (0.011)	0.952 (0.013)	0.855 (0.018)	0.841 (0.025)
**Legal Recognition**				
Preferred Gender is on IDs	---	0.883 (0.122)	1.023 (0.147)	1.035 (0.255)
Preferred Name is on IDs	---	1.041 (0.139)	0.925 (0.133)	0.835 (0.208)
**Experience and Treatment Barriers**				
Safety	0.514 (0.049)	0.495 (0.118)	---	---
Mistreatment	0.893 (0.025)	0.984 (0.062)	0.899 (0.040)	0.895 (0.043)
Bad experiences	0.815 (0.030)	0.793 (0.083)	0.813 (0.046)	0.726 (0.054)
Provider Not Comfortable Caring for Transgender Patients	0.862 (0.028)	0.852 (0.075)	0.911 (0.041)	0.764 (0.054)
**Logistic Barriers**				
Time	0.640 (0.048)	---	---	0.448 (0.074)
Cost	0.644 (0.052)	---	---	0.553 (0.072)
Inconvenience	0.767 (0.049)	---	---	0.561 (0.068)
**Positive Healthcare Experiences**				
Satisfaction	0.855 (0.060)	---	0.786 (0.109)	0.768 (0.075)
My provider is knowledgeable about health issues facing transgender persons.	0.627 (0.050)	---	0.624 (0.093)	0.734 (0.064)
Have Provider	---	---	0.452 (0.099)	0.709 (0.071)

^1^Fit statistics can be found in supplemental Table 2

^^^Pride and Community Connectedness questions were not asked among the online mode participants

### Structural equation models

**Fig. 2:**
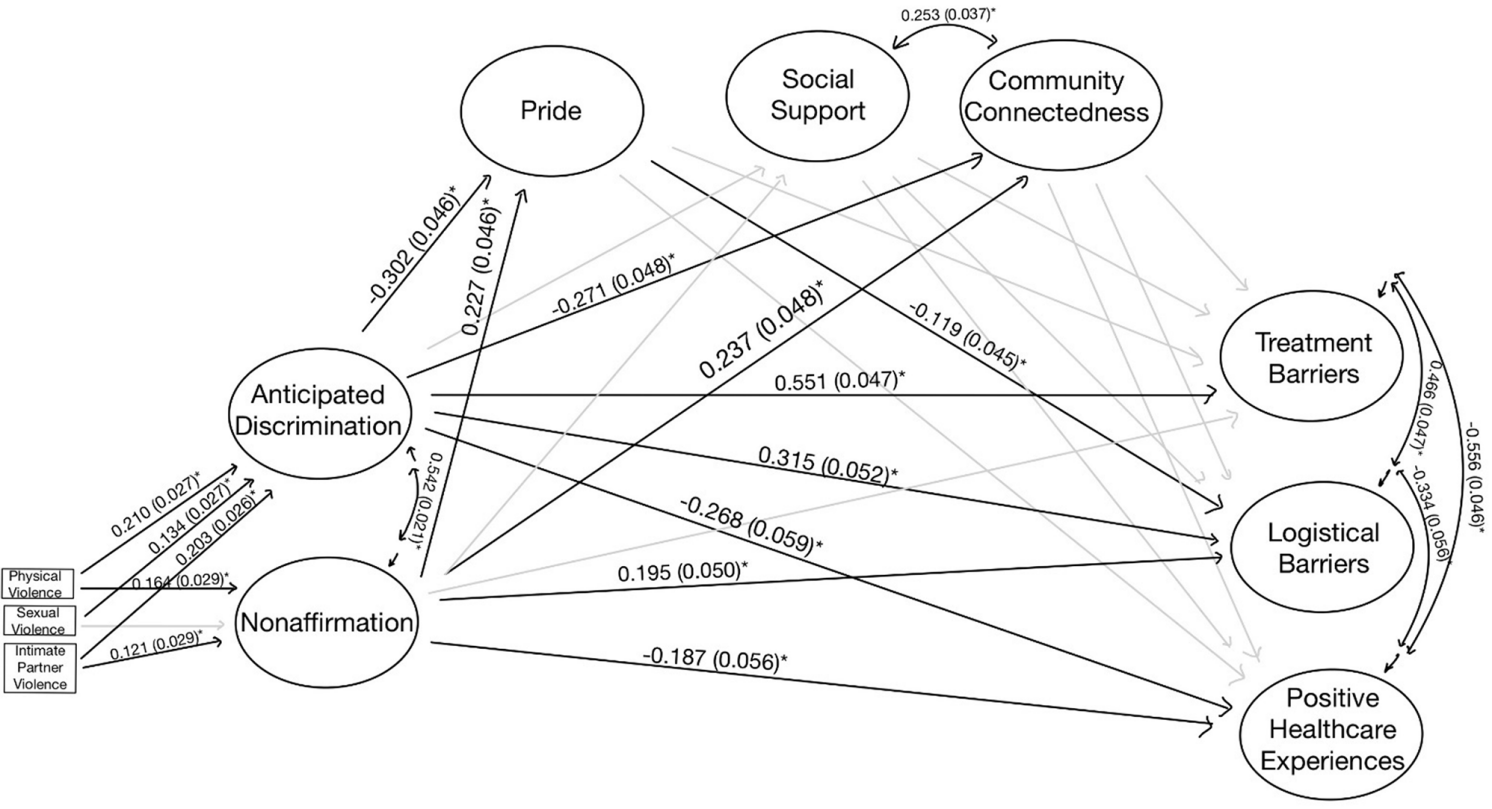
Entire Sample– SEM (*p < 0.05). Fit statistics for this model are included in Supplemental Table 3

**Fig. 3:**
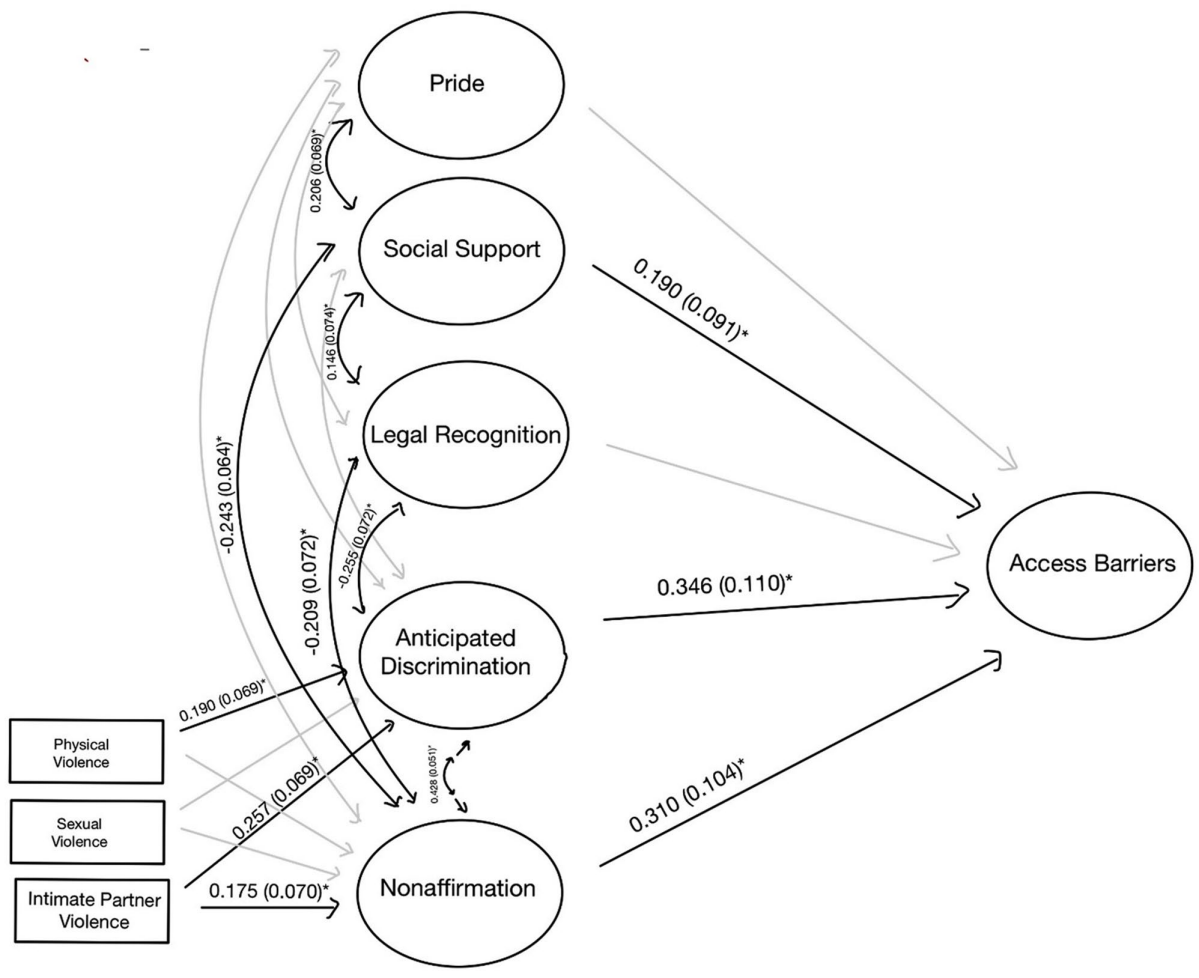
TW Living with HIV – SEM (* *p* < 0.05). Non-mediated model of the association between gender minority stress and resilience factors with access barriers. Fit statistics for this model are included in Supplemental Table 3

**Fig. 4:**
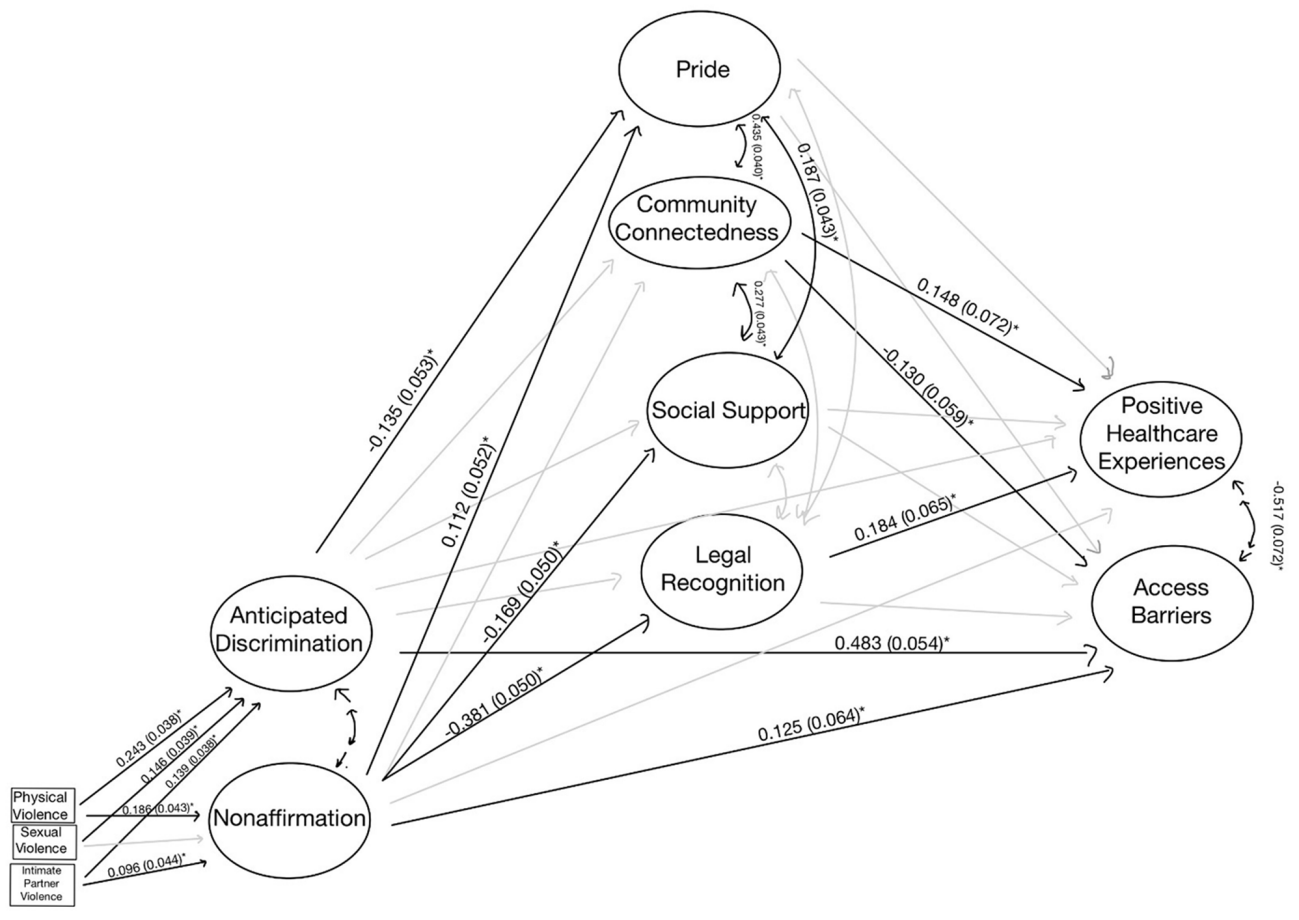
TW Living without HIV [Site-Based] – SEM (* *p* < 0.05). Fit statistics for this model are included in Supplemental Table 3

**Fig. 5:**
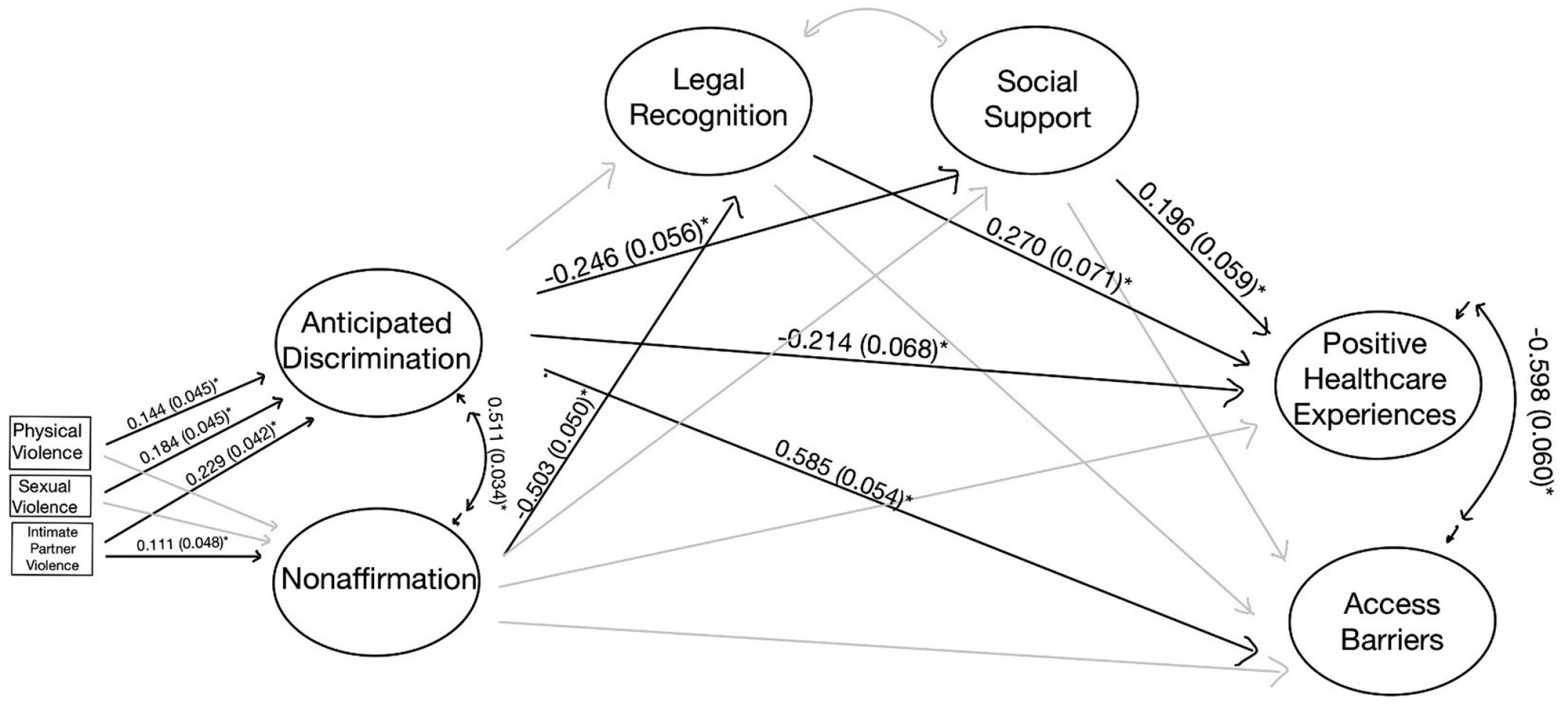
TW Living without HIV [Online] – SEM (* *p* < 0.05). Fit statistics for this model are included in Supplemental Table 3

In the mediated models, indirect pathways between gender minority stress and access to care were statistically significant for the entire sample and both the site-based and online groups living without HIV. Thus, the unadjusted, mediated models were presented in Figs. 2, 4, and 5 for these analytic samples. For the TW Living with HIV group, the estimates and fit did not differ between adjusted and unadjusted models, and thus the unadjusted, unmediated model is presented in Fig. 3. All models exhibited good fit (Supplemental Table 3).

Among the entire sample, higher anticipated discrimination and non-affirmation were associated with fewer positive healthcare outcomes and increased treatment and logistical barriers (Fig. 2). Gender pride mediated the associations between non-affirmation and logistical barriers and between anticipated discrimination and logistical barriers. Accounting for the mediating relationship, the indirect effect of non-affirmation and logistic barriers was − 0.045 (0.021, *p* < 0.05) and the total was 0.150 (0.045, *p* < 0.05), indicating that a higher level of non-affirmation was associated with increased logistic barriers, but this was slightly attenuated by increased gender identity pride. However, the indirect effect between anticipated discrimination and logistic barriers was 0.053 (0.023, *p* < 0.05), and the total effect was 0.368 (0.044, *p* < 0.05). This indicates that the relationship between anticipated discrimination and logistic barriers increased with increased gender pride. Non-affirmation was positively associated with community connectedness and gender pride, with a direct effect of 0.237 (0.048, *p* < 0.05) and 0.227 (0.046, *p* < 0.05), respectively.

Among TW living with HIV, there was no significant mediation by resilience factors of the relationship between gender minority stress and access to care (Fig. 3). Those with higher levels of anticipated discrimination and non-affirmation experienced more barriers to healthcare access. Social support did not appear to affect the correlation between minority stress and access to care, as anticipated discrimination and non-affirmation were still associated with increased barriers to healthcare access among those with higher levels of social support (direct effect: 0.190 (0.091, *p* < 0.05)).

Among the site-based sample of TW living without HIV, legal recognition of gender identity attenuated the relationship between non-affirmation and positive healthcare experiences, with a total indirect effect of -0.073 (0.029, *p* < 0.011) (Fig. 4). The direct relationship between non-affirmation and positive healthcare experiences was not statistically significant (*p* = 0.060), and the total effect was − 0.202 (0.064, *p* < 0.05), meaning that those with higher levels of non-affirmation reported fewer positive healthcare experiences, though legal recognition attenuated this effect. Neither non-affirmation nor anticipated discrimination were directly associated with positive healthcare experiences, while both were positively associated with barriers to healthcare access. Gender pride was not directly associated with positive healthcare experiences nor barriers to healthcare access; however, those with increased anticipated discrimination experienced lower levels of gender pride, while those with increased levels of non-affirmation experienced higher levels of gender pride.

Among the online mode participants living without HIV, non-affirmation was not significantly associated with positive healthcare experiences, barriers to healthcare access, nor social support (Fig. 5). Nevertheless, those with higher levels of non-affirmation experienced lower levels of legal recognition. Legal recognition affected the association between non-affirmation and positive healthcare experiences, with an indirect effect of -0.136 (0.040, *p* < 0.05), though the total effects are not statistically significant. Additionally, social support impacted the association between anticipated discrimination and positive healthcare experiences, with an indirect effect of -0.048 (0.017, *p* < 0.05). The total effect was − 0.269 (0.066, *p* < 0.05), indicating that those with higher levels of anticipated discrimination experience decreased positive healthcare experiences.

## Discussion

Overall, in this study of TW living with and without HIV and residing in the eastern and southern United States, higher levels of gender minority stress were found to be associated with decreased access to healthcare, most notably increased logistical and treatment barriers, as well as decreased positive healthcare experiences. Among both TW living with and without HIV who participated in the site-based mode, anticipated discrimination and non-affirmation were also associated with increased barriers to healthcare access. Among TW living without HIV in the online mode, anticipated discrimination was associated with barriers to healthcare access, though non-affirmation was not. These findings align with prior research, which found that greater anticipated stigma was associated with less use of gender affirming care among transgender youth [11], and that both logistic barriers to care and negative experiences in healthcare became a subsequent barrier for young TW living with HIV [37]. Repeatedly having to correct others on their gender identity was not associated with non-affirmation for the site-based analytical group. This could contribute to the relationship between non-affirmation and barriers to healthcare access, as these are well-established facets of resilience [16].

We observed significant direct associations between resilience and access to care, with resilience associated with positive healthcare experiences and inversely associated with barriers to healthcare access. Among TWLHIV, those with higher levels of social support experienced increased barriers to healthcare access. Social support was also negatively correlated with non-affirmation, which may indicate that TW who experience increased levels of non-affirmation may not seek social support or that TW who have higher social support experience lower levels of non-affirmation, though this did not affect the positive association between non-affirmation and barriers to healthcare access.

Among the site-based group of TW living without HIV, community connectedness was associated with positive healthcare experiences and fewer barriers to healthcare access. This finding suggests that higher resilience may result in fewer barriers and increased positive experiences in treatment. In a study of transgender youth, community connectedness was found to help individuals navigate healthcare systems and increase access [11]. A systematic review also found community connectedness among transgender individuals was associated with increased healthcare access in three studies [2]. Additionally, one barrier to having care is finding providers that are experienced in caring for transgender patients, so experiencing higher levels of community connectedness and social support may result in increased resources to know who these providers are and how to find appropriate and competent care [38].

Most of the resilience factors were not found to mediate the relationship between gender minority stress and access to care, though there were notable exceptions. Across all TW analytical groups, non-affirmation was positively associated with community connectedness and pride. This may indicate that TW who experience higher levels of non-affirmation seek a sense of community and relatedly experience an enhanced sense of gender pride. However, the resilience factors did not have significant indirect effects related to the association between gender minority stress and access to care in these analytical groups. One key resilience indicator that did mediate the relationship between non-affirmation and positive healthcare experiences in TW living without HIV in the site-based mode was legal recognition of gender identity. Often, patients must give legal name and sex assigned at birth to medical providers, which may lead to misgendering and possible non-affirming experiences. More than one-third of the LGBTQ population live in states that require publication of a name change announcement, and 45% live in a state where it is difficult to understand forms to change sex on legal identification or requires provider certification [39]. Reducing barriers to legal gender recognition could indirectly improve healthcare access for TW. Further, developing infrastructure within clinical settings to allow patients to report their chosen name and gender within medical records, as recommended by the US CDC [40], may reduce gender non-affirmation and increase access to healthcare for transgender patients [41].

Generally, the results of the present study do not align with the mediating effect of resilience in minority stress theory [27]. One reason for this is simply because this study examines healthcare access as its outcome, as opposed to physical and mental health outcomes that are the focus in the original framework. It is possible that resilience may be a mediator in the relationship between minority stress and physical and mental health outcomes, but not in the relationship with access to care.

The safety indicator used to measure healthcare access was one of the most noticeable sources of measurement invariance between the three analytic groups. Concerns about safety in transit to healthcare was only significant for the access to healthcare construct among TWLHIV, indicating that they are concerned about safety when accessing healthcare compared to TW living without HIV. Violence is a well-documented correlate of HIV seroconversion [42], has been associated with disclosure of HIV status, and TW experience some of the highest levels of violence, including community violence. The regular healthcare visits required for HIV care likely increase potential exposure to violence in transit to care; thus, TWLHIV likely weigh the risk of violence against the benefit of health services when making decisions about healthcare utilization. In this sample, 97.6% of the TWLHIV were linked to HIV care, but 82.5% did not report having a regular personal healthcare provider. Currently, Ryan White funding has been used to provide medical transportation for patients living with HIV who qualify for financial support, and these have been used to provide transportation vouchers, ride services, and support telehealth to reduce transportation-related barriers to care [43]. However, expanding transportation services to others who do not qualify for Ryan White support or who are not living with HIV may reduce barriers to healthcare for transgender women.

Contrary to our expectations and findings in the current literature on TW’s access to care [1], health insurance was not found to be a significant factor for healthcare access among any of the analytic groups. Health insurance is included in most theoretical frameworks on access, and in much of the current literature, it is used as a proxy indicator for healthcare access [9, 44]. However, the importance of health insurance as an indicator of access may differ based on HIV status, due to the Ryan White funding to support care among individuals living with HIV. It is also possible that this is a limitation of our sample, as less than 10% of each analytic group reported being uninsured, though this may be attributed to recent policy changes such as Medicaid expansion that varies by state.

As discussed above, we observed some relationships among site-based mode participants that were not observed among digital mode participants. Specifically, the following relationships among site-based participants that were not observed among digital mode participants: (1) experiences of non-affirmation were associated with increased barriers to healthcare access among site-based participants, (2) and legal recognition of gender identity mediated the relationship between non-affirmation and positive healthcare experiences. This may be explained in part by the different characteristics of site-based and online mode participants and may reflect the “digital divide” in terms of social and structural factors that influence who has consistent access to technology to participate in research. For example, participants in the site-based mode were more likely to have public insurance and may be more restricted in where they can access health care, while those in the online mode may have increased access to telehealth as well as improved access to providers through private insurance. Thus, for site-based participants, historical experiences of non-affirmation may result in feeling like there are increased barriers to healthcare. As healthcare and technology continue to co-evolve, it will be important to continue to assess how this affects healthcare for individuals with inconsistent technology or internet access.

### Limitations

There were several limitations to our analyses. First, the analytic groups (mode and HIV status) were not directly comparable due to heterogeneity in demographics and other characteristics related to minority stress and resilience. The differences in results of the SEM across the analytic groups may be attributed to differences in the experiences of non-affirmation and experience and treatment barriers to healthcare. However, findings may reflect the unique pathways identified in these two samples or may reflect the differences in each sample, particularly in those that access clinical sources versus digital-based resources [45]. We also did not note any significant confounding by race in the SEM, though this may also reflect racial heterogeneity between analytical groups. It is possible that there would be differences in healthcare access by race or ethnicity, and future research should examine this potential impact, as well as the necessity of cultural competence when delivering care to transgender women of color.

Additionally, elements of online social support, community connectedness, and gender pride were not measured for the online analytical group. However, the analytic groups showed similar associations between the latent constructs, although significant measurement invariance was identified across groups. An important direction for future research is to examine the extent to which the associations may differ by HIV status, possibly due to intersectional stigmas experienced related to both HIV status and being transgender [46]. HIV-related stigma has been shown to be associated with decreased health service utilization [47]. Additionally, more research is needed about how transgender women access online resources for healthcare.

Second, general health was defined in this study to be health unrelated to gender affirmation or transition, but this was not explicitly stated in the questions. Although there were additional questions in the survey related to gender-affirming care specifically, participants may have interpreted questions on access barriers to include gender-affirming care.

Third, as a cross-sectional analysis, causality and temporality could not be assessed, limiting our inferences regarding whether resilience mediates the relationship between gender minority stress and access to healthcare and whether there is potential for reverse causation. However, these findings pointed to potential avenues of importance to explore in future longitudinal research.

The site-based analytical groups were affiliated with clinics that serve the transgender community, so the relationships demonstrated in these groups may be attenuated compared to populations that do not have this kind of access. Lastly, there was poor measurement model fit among the access to care factors among the online group that was not living with HIV, which may obscure the results of the structural model for that group. Numerous indicators of healthcare access could differ among TW who have high utilization of technology-based services, such as the use of telemedicine, online social support and mental health support, digital pharmacies, and digital health literacy. Future research in this field should identify the structural and interpersonal causes of gender minority stress to inform interventions to ameliorate such effects on healthcare access.

The limitations are lessened by the strengths of the study. This cohort was one of the largest samples of TW in the US, covered a vast region of the eastern and southern US, and used several measures validated for the transgender population (e.g., measures of intersectional discrimination, various resilience indicators, intimate partner violence, etc.). Use of mixed cohort modalities, along with these strengths, enhance generalizability to the population of TW in the US.

## Conclusion

This study showed that gender minority stress is positively associated with barriers to accessing general healthcare among TW in the US, regardless of HIV status. However, resilience factors were less uniformly associated with healthcare access and suggested that resilience factors supporting healthcare access for transgender women may vary depending on whether they are living with HIV. Legal gender recognition was shown to mediate the relationship between non-affirmation and positive experiences among those living without HIV, suggesting a possible structural avenue for improving access, including modernizing electronic medical records to permit use of patients’ gender and chosen name. Nevertheless, our findings did not support resilience as mitigating the effects of gender minority stress on healthcare access, suggesting that while forms of resilience are important to recognize and promote, public health interventions must directly mitigate gender minority stress to increase access to general healthcare for TW in the United States. In particular, the strong relationship between mistreatment by healthcare workers and perceived discomfort by healthcare workers in caring for transgender patients and access to care indicates that cultural competency training in health facilities is of utmost public health importance. This can and should include both interventions that address provider and staff knowledge and attitudes, but also structural level policy initiatives that seek to make care more accessible and more culturally competent, such as integration of more transgender health and assessment of implicit biases into medical education.

### Electronic supplementary material

Below is the link to the electronic supplementary material.

**Supplementary Material 1:** Consortium information and supplemental tables

## Data Availability

The datasets used and analyzed during the current study are available from the study team (Andrea L. Wirtz) on reasonable request.

## Abbreviations

TW: transgender women
HIV: Human Immunodeficiency Virus
US: United States
HRSA: Health Resources and Services Administration
LITE: Leading Innovation for Transgender Women’s Health and Empowerment Study
TWLHIV: transgender women living with HIV
EFA: exploratory factor analysis
CFA: confirmatory factor analysis
RMSEA: root mean-square error of approximation
SRMR: standardized root mean square residual
TLI: Tucker-Lewis fit index
CFI: comparative fit index
SEM: structural equation modeling
IQR: Interquartile range
LGBTQ: Lesbian, Gay, Bisexual, Transgender, Queer/Questioning
