# Supplementary material for: Access to healthcare among transgender women living with and without HIV in the United States: associations with gender minority stress and resilience factors
Source: BMC Public Health. 2024 Jan 20;24:243. doi: 10.1186/s12889-024-17764-y (PMC10800069; doi:10.1186/s12889-024-17764-y)
Supplement: Supplementary file 1 — Supplementary Material 1: Consortium information and supplemental tables [file 12889_2024_17764_MOESM1_ESM.docx]

*Supplement*

**American Cohort to Study HIV Acquisition Among Transgender Women Study Group Members and the LITE Community Advisory Board**

Members of The American Cohort to Study HIV Acquisition Among Transgender Women (also known as the LITE cohort) include Sari Reisner (principal investigator; Brigham and Women’s Hospital); Andrea Wirtz (principal investigator; Johns Hopkins University); Keri Althoff (Johns Hopkins University); Chris Beyrer (Johns Hopkins University); James Case (Johns Hopkins University); Erin Cooney (Johns Hopkins University); Meg Stevenson (Johns Hopkins University); Dee Adams (Johns Hopkins University); Oliver Laeyendecker (National Institute of Allergy and Infectious Diseases); Charlotte Gaydos (Johns Hopkins University); Tonia Poteat (University of North Carolina); Kenneth Mayer (Fenway Health); Asa Radix (Callen-Lorde Community Health Center); Christopher Cannon (Whitman-Walker Health); Jason Schneider (Emory University and Grady Hospital); J Sonya Haw (Emory University and Grady Hospital); Allan Rodriguez (University of Miami); Andrew J Wawrzyniak (University of Miami); the LITE Community Advisory Board: Sherri Meeks, Sydney Shackelford, Nala Toussaint, SaVanna Wanzer, and others who have remained anonymous.

**Supplemental Table 1.** Reliability for Scales

| Scale | Cronbach’s Alpha |
| --- | --- |
| Pride | 0.8495 |
| Social Support | 0.8855 |
| Community Connectedness | 0.7294 |
| Non-affirmation of gender identity | 0.9106 |
| Discrimination | 0.9085 |
| Physical Violence | 0.8753 |
| Sexual Violence | 0.8609 |
| Intimate Partner Violence | 0.8510 |
| Intimate Partner Violence, last year | 0.8542 |

**Supplemental Table 2**. Measurement Model Fit Statistics

|  |  | **Chi-Square** | **RMSEA (Upper Limit)** | **SRMR** | **CFI** | **TLI** |
| --- | --- | --- | --- | --- | --- | --- |
| **Entire Sample** | **Gender Minority Stress** | p<0.05 | 0.095 (0.102) | 0.040 | 0.973 | 0.967 |
|  | **Resilience** | p<0.05 | 0.097 (0.103) | 0.164 | 0.909 | 0.876 |
|  | **Access to Care** | p<0.05 | 0.072 (0.084) | 0.065 | 0.953 | 0.930 |
| **Living with HIV** | **Gender Minority Stress** | p<0.05 | 0.095 (0.115) | 0.053 | 0.976 | 0.971 |
|  | **Resilience** | p<0.05 | 0.081 (0.097) | 0.103 | 0.974 | 0.970 |
|  | **Access to Care** | 0.8109 | 0.00 (0.103) | 0.016 | 1.00 | 1.00 |
| **Living without HIV [site-based]** | **Gender Minority Stress** | p<0.05 | 0.100 (0.112) | 0.047 | 0.971 | 0.971 |
|  | **Resilience** | p<0.05 | 0.082 (0.090) | 0.077 | 0.972 | 0.970 |
|  | **Access to Care** | p<0.05 | 0.088 (0.121) | 0.069 | 0.970 | 0.943 |
| **Living without HIV [online]** | **Gender Minority Stress** | p<0.05 | 0.097 (0.111) | 0.049 | 0.959 | 0.950 |
|  | **Resilience** | p<0.05 | 0.076 (0.116) | 0.021 | 0.994 | 0.989 |
|  | **Access to Care** | p<0.05 | 0.127 (0.147) | 0.115 | 0.830 | 0.764 |

**Supplemental Table 3**. Structural Model Fit Statistics

|  | **Chi-Square** | **RMSEA (Upper Limit)** | **SRMR** | **CFI** | **TLI** |
| --- | --- | --- | --- | --- | --- |
| **Entire Sample** | p<0.05 | 0.053 (0.055) | 0.083 | 0.935 | 0.929 |
| **Living with HIV** | p<0.05 | 0.043 (0.049) | 0.080 | 0.974 | 0.972 |
| **Living without HIV [site-based]** | p<0.05 | 0.044 (0.047) | 0.059 | 0.965 | 0.962 |
| **Living without HIV [online]** | p<0.05 | 0.054 (0.058) | 0.065 | 0.953 | 0.947 |
